# Direct to consumer genetic testing and gamete donor conception: ethical challenges

**DOI:** 10.1186/s12910-026-01449-9

**Published:** 2026-04-11

**Authors:** Lucy Frith

**Affiliations:** Centre for Social Ethics and Policy, Department of Law, University of Manchester, The Williamson Building, Oxford Road, Manchester, M13 9QQ UK

**Keywords:** Eggs and sperm, Gamete, Donation, Anonymity, Reproductive technologies, Information giving, Third party reproduction, Direct to consumer, Genetic DNA testing

## Abstract

This paper examines recent developments in direct-to-consumer genetic (DNA) testing (DTCGT) and the ethical challenges it raises for those affected by donor conception (using donated gametes, eggs and sperm, to conceive children). First, DTCGT can result in the complete removal of donor anonymity at any stage in the donor conceived person’s life, and this has widespread implications for all those involved in donor conception. Second, for regulators: DTCGT can disrupt regulatory prohibitions on information disclosure. Third, for donor conceived people: there is potential harm in discovering you are donor conceived via DTCGT, and DTCGT creates new 'gatekeepers' of knowledge about donor conception, who have to decide whether to disclose this information. DTCGT also raises normative questions for those involved in donor conception. For donors: do donors have any obligation to participate in DTCGT, or to provide their genetic data to donor conceived offspring? For parents through donor conception: how does DTCGT affect decisions about disclosing donor conceived origins to their child, and should parents test their child, and if they decide to do so, when? The paper concludes by making recommendations for how the ethical challenges posed by DTCGT might be overcome or at least ameliorated.

## Introduction

Reproductive donation, the donation of gametes, eggs and sperm and, if combined, embryos to others to have children, has been one of the more controversial of assisted reproduction techniques. It has led to ongoing, and yet unresolved debates, about what information donor conceived people should be given about their donor and if they have a right to, or a significant interest in, this information. There are also questions of what information should be shared between donor-siblings (people conceived from the same donor)? And who should act as a gatekeeper of this information, a national registry, fertility clinics or individuals? These questions have been debated ever since artificial insemination with donor sperm became a topic of medical discussion over 70 years ago [1]. The increasing use of direct-to-consumer genetic (DNA) testing (DTCGT) and online social media platforms have changed the landscape in which donor conception operates. There are now new and unanticipated opportunities to find information that can identify the donor, donor-siblings and wider donor relatives.

This paper will examine the ethical challenges raised by the rise in the use of DTCGT, by considering how it affects regulators, donor conceived people, donors and parents by donor conception. First, DTCGT could result in the complete removal of donor anonymity at any stage in the donor conceived person’s life, and this has widespread implications for all those involved in donor conception. Second, for regulators, DTCGT has the potential to disrupt regulatory prohibitions on information giving in donor conception. Third, for donor conceived people, it can be harmful to find out you are donor conceived via a DTCGT site. DTCGT also creates new 'gatekeepers' of knowledge about donor conception, who have to decide whether to disclose this information. Further, DTCGT raises normative questions for those involved in donor conception. For donors, do they have an obligation to participate in DTCGT, or to provide their genetic data to any donor conceived offspring? For parents through donor conception, how does DTCGT affect decisions about disclosing donor conceived origins to donor conceived offspring and should parents test their child and, if so, at what age? The paper concludes by making recommendations for good practice so that the ethical challenges posed by DTCGT might be, if not overcome, at least ameliorated.

The ethical challenges discussed here are not exhaustive, and no doubt there are others. DTCGT has not ‘created’ these issues, nor does it determine any particular outcome, but it changes the context for existing ethical questions posed by donor conception, such as how to approach disclosure of donor conceived origins to donor conceived offspring, and gives rise to new ones, such as if, and when, to do a DTCGT for your child. The ethical challenges discussed here have been derived from empirical projects on donor conception and DTCGT, such as the ConnecteDNA project [2, 3], and a consideration of the ethical and practice literature. As the interaction between DTCGT and donor conception becomes studied more thoroughly other questions and issues will no doubt present themselves, but for the purposes of this paper I shall concentrate on what, currently, I take to be the most pressing issues.

Terminology for individuals involved in donor conception is contested, bodies such as The American Society of Reproductive Medicine (ASRM) have recommended terminology change in this area,^1^ and concepts such as anonymity and the categories of information held by official bodies require clarification. To address this, Table 1 sets out the terminology and the definitions of approaches to information giving in reproductive donation that are used in the literature. The debate over donor anonymity in the literature is largely conducted in a northern American, European and Australian context and within Euro-American kinship framings. Therefore, the issues raised by DTCGT may be different in other geographical and cultural contexts.

**Table 1:** Terminology

TERM	DEFINITION
**Types of people involved in donor conception**	
Recipient/s	Person or persons who are receiving the gamete to become parents and will become the legal parents of any resulting child. Recipients can be couples or individuals
Donor-conceived person	A person conceived via the use of donated gametes – egg and/or sperm
Donor-conceived offspring	A donor-conceived person under the age of 18
Donor-sibling	A genetic half-sibling, conceived from the same donor
Donor’s ‘own’ children	People conceived by the donor, not in their capacity as a donor
Donor relative	Those related to someone through donor conception (the donor, donor sibling, extended family of the donor). These relationships do not have to be genetic, i.e., the partner of the donor could be a donor relative
**Types of information**	
Non-identifying information	Information that does not identify the donor: details of the donor’s physical appearance, interests and education. In the UK, this is provided by donors on the donor registration form, in what are called pen portraits, or information provided to a sperm or egg bank
Identifying information	Information, as the name suggests, is information that could identify the donor such as their name, last known address or other contact details
**Types of donation**	
Anonymous donation Non-identified donors	The donor is anonymous to any future donor-conceived person and the recipients. The clinic act as the intermediary between recipients and the donor during treatment The ASRM [4] uses this term to refer to donors who at the time of donation are not known to the recipients and their identity is planned to remain unknown to the donor conceived person
Information release	Recipients and donor conceived people can find out non-identifying information about the donor, and the donor can find out non-identifying information about their donation, i.e. how many children were born and what gender
Known donation Directed donation	The recipient/s bring a donor to the clinic for their use. Or this could be done informally in the case of sperm donation outside of a clinic The ASRM [4] has suggested that known donation should be known as ‘directed donation’
Identity release	The donor is initially anonymous, but their identity can be released to the donor-conceived person when they reach a certain age, i.e., an age of majority such as 18
**Regulatory approaches**	
Mandatory anonymous donation	Jurisdictions do not allow the giving of identifying information to the donor-conceived person
Mandatory information recording	Jurisdictions mandate the collection of information about the donor and recipients usually at clinic or registry level
Mandatory information release	Information is allowed to be given to donor-conceived people, recipients and donors, if requested. This can be non-identifying or identifying information depending on the regulatory framework, and the timing of this depends on jurisdiction
Registries	Jurisdictions hold registries of information about the donor, recipients and outcomes of treatment. Examples of these are the register held by the Human Fertilisation & Embryology Authority in the UK and in the Victoria, Australia, what was the Victorian Assisted Reproductive Treatment Authority
Double track	Jurisdictions that allow both anonymous and non-anonymous donation [5]

This paper contributes to the debates over information giving in donor-conception by situating them in the new landscape in which donor conception now operates. Many of the past strictures surrounding information provision, such as the imperative to keep it secret from the donor conceived person, wider family and friends [6], were due to societal attitudes towards donation, its unregulated nature and legal uncertainties over parenthood [7]. These concerns are no longer so relevant, and the debates over information giving in reproductive donation must respond to this new era of more easily available information and increased possibilities for contact between donor relatives. DTCGT reinvigorates theoretical questions about the significance of genetic origins for identity formation and raises new practical ethical challenges. Addressing these challenges is timely, as this new landscape has prompted legislators, both in the UK and internationally, to consider what, if any, regulatory response is needed [8–11].

## Background

### Information giving in gamete donor conception

The debates over the use of DTCGT need to be contextualised within the long-running debate over information provision in donor conception. Should the donor be anonymous to any future offspring or should donor programmes release the identity of the donor to the donor conceived person at some specified time, i.e., the age of majority? And should parents tell their child they are donor conceived, and if so, when? As Francois Shenfield said over 30 years ago, information giving is the ‘main controversy in assisted reproduction with donor gametes’ [12], 371). Groll calls whether the donor should be known or anonymous to future offspring the ‘central question’ in donor conception [13]. There are two levels at which this debate takes place. The first is the regulatory level where legislation, professional guidelines or clinic policies operate, depending on the jurisdiction, which determine whether donation is anonymous or identity-release and how information is stored, who can access it and how it is safeguarded. The second, is the family level, how information about donor conception is managed within families, when and how disclosure occurs, at what age, and the potential consequences of non-disclosure.

The debates over whether donors should be anonymous or identity-release and whether to tell children they are donor-conceived are still live debates, and little consensus has been reached. *Fertility and Sterility*, the journal of the ASRM, ran a debate on the topic in 2025 [14] and donor anonymity is included in the textbook *50 Big Debates In Reproductive Medicine* published in 2021 [15]. Further evidence of the lack of consensus over whether gamete donors should be anonymous or identifiable to their donor offspring, is the significant regulatory variation across different jurisdictions on this issue [16]. There are countries that have identity-release donation. In the UK, for example, donor conceived people can access identifying information about the donor from the age of 18 and in the Netherlands at 16. In other countries donation is done under conditions of anonymity, the donor conceived person has no rights to access identifying information about their donor at any age, countries such as Spain and Italy operate under these conditions. In Spain, although the donor is anonymous, the Spanish Fertility Society recommends that child should be told they are conceived from donor gametes [17]. Further, there are countries that allow both anonymous and identity-release donation, such as Denmark and Greece. The US has state-based legislation and depending on the state and clinic both identity release and anonymous donation are available [18].

Arguably, part of the reason for this lack of consensus is that information giving in reproductive donation raises profound ethical and philosophical questions about what it means to be a parent and the importance of genetic origins. A central issue is what rights or interests people have in knowing their genetic origins. In donor conception this is the question of how important is it for people to know that they are donor conceived and subsequently be able to access information about and, possibly, the identity of the gamete donor? And what, if any, duties do the donor conceived person’s interests or rights to information place on others? This speaks to what information is important and meaningful to people, how we view and construct the family and what family forms are supported both socially and legally. In this section I will outline this debate and Table 2 provides further details of the main points and protagonists.

**Table 2:** Views on donor anonymity

Arguments against anonymity	Summary of position	Authors/citations
Against gamete donor anonymity	Anonymity is seen as an unacceptable way of organizing reproductive donation	Eric Blyth Vardit Ravitsky Naomi Cahn
**Practical arguments**		
Better for the donor conceived person	Access to health information is useful for the donor conceived person Access to information is useful for identity forming information for the donor conceived person Reduces small risk of consanguinity	[19] [20]
Better for family functioning	Recipients have information to give their donor-conceived offspring about the donor Non-anonymity encourages openness and not keeping donor conception secret, which is better for family functioning	[21] [22]
Anonymity can no longer be guaranteed due to DTCGT	Pragmatically anonymity can no longer be guaranteed. Hence, greater risks of donor conceived people finding out accidently they are donor conceived	[23] [3]
**Principled arguments**		
Right of the donor-conceived person to know their genetic origins	This is sometimes argued to be a human right, under the United Nations' Convention on the Rights of the Child (1989) has as Article Seven, the right to know one's parents	[24] [25] [26]
Significant interest of the donor conceived person to know their genetic origins	Donor-conceived people are likely to have a ‘significant interest’ in knowing who the donor is. ‘Parents ought to promote their children’s well-being by helping satisfy their child’s worthwhile significant interests, and a donor-conceived person’s interest in genetic knowledge is one such interest.’	[13]
Anonymity restricts a donor conceived person’s choices	Identity-release donation gives donor-conceived people the choice over the importance they want to give to information about their genetic origins, a choice most people have	[27]
**Implications of these views**		
Views on disclosure	Generally, those who favour non-anonymity favour disclosure of donor-conceived origins Disclosure is important, and secrecy problematic	[28] [29] [30]
Genetic knowledge	Genetic knowledge is seen as important	[31]
Identifying information about the donor	Importance for the donor conceived person having identifying information about the donor	[31] [13]
Identifying information about	Importance of donor conceived person finding donor-siblings, and other donor relatives	[32]

**Arguments for anonymity**	**Summary of position**	**Authors/citations**
Anonymity is an acceptable way of organizing reproductive donation	Anonymity is an acceptable option, and it could be offered alongside other non-anonymous forms of donation	Guido Pennings I Glenn Cohen Inmaculada De Melo-Martin
**Practical arguments**		
It will preserve donor numbers	Removing donor anonymity will reduce the numbers of donors	[33, 34] [5]
Maintenance of donor supply enables more people to have fertility treatment	If donor numbers are reduced it will restrict the number of people who can access reproductive donation	[33]
Best for the family	Reduce stigma Prevents unwanted contact Protects the integrity and stability of the family Maintains privacy Keeps legal parenthood separate from genetic parenthood	[35] [36]
Best for the child	They are brought up in a stable family and there is no intrusion of the donor into these family relationships	[6]
Better for the donor	Protects them against unwanted parental claims	[37]
Better for the clinics	Enables the operation of donation programmes more easily	[1, 38]
**Principled arguments**		
There is no right to know one’s genetic origins	There is no right as it is based on unwarranted assumptions about the importance of genetic origins	[39]
Respects the autonomy of recipients	Their right to choose to have an anonymous donor	[34]
Respect for autonomy of the donor	Their right to donate anonymously	[5]
Cultural and religious reasons	Some cultures have views on inheritance and that genetic parenthood cannot be transferred. In some communities fertility treatment is highly stigmatised, anonymity enables it to be concealed	[40] [41]
**Implications of these views**		
Views on disclosure	Benefits of knowing are negligible	[15]
On importance of genetic knowledge	Genetic knowledge is not seen as particularly important	[42]
All parties’ rights need to be balanced	The respective parties, donor-conceived people, the donor, recipient parent(s), rights or interests should be balanced, and donor conceived peoples’ rights or interests do not trump other parties’ rights	[43] [44]
Allows both anonymous and non-anonymous donation	Many of the arguments for anonymity do not stipulate it should be the only choice. If no agreement on the ‘best’ way, have a double track so recipients and donors can choose Allows recipients and donors choice over how they participate in reproductive donation	[5]

The debate in the ethical and philosophical literature is polarised between those who see anonymous gamete donation either as an acceptable moral choice or the preferrable one, and those who argue for non-anonymity, identity-release donation, and greater openness and disclosure of donor conception origins to donor conceived people. There are few who now argue that anonymous donation is the only acceptable choice. Rather, the claim is that anonymity is an acceptable option, and it should be offered alongside other non-anonymous forms of donation [15]. The arguments in the ethical literature over whether gamete donors should be anonymous to any future offspring are a mixture of practical and principle-based considerations.

Practical arguments against anonymous donation concern the potentially harmful consequences of the donor being anonymous to the donor conceived person and how it affects family functioning [21] and these are often framed as empirical claims. The central principle-based argument against donor anonymity, is that people have a fundamental right or interest to know their genetic origins. The claim is that knowledge of genetic parents is important, and that it is either a right (of some kind) or an important interest, and any systematic denial of this potentially harms and/or wrongs donor conceived people and is therefore unethical. Ravitsy argues that the right to know,

‘is grounded, not necessarily in a need for protection from harm, but rather in their autonomy to make choices about what their genetic origins mean to them, at different points in their lives. This choice is linked to fundamental aspects of human existence: our understanding of who we are and how we are connected to others.’ ([27], 36)

This has been argued to be a human right (see [45, 46]. The United Nations’ Convention on the Rights of the Child Article Seven gives children the right to know their parents. In UK case law, it was acknowledged that Article 8 of the European Convention for the Protection of Human Rights and Fundamental Freedoms was engaged with regard to identifying and non-identifying information about a genetic parent [47]. Constructing access to identifying information about the donor as a human right that should be legally enforceable has underpinned some jurisdictions’ rationale for the removal of donor anonymity [48].

There are other ways of seeing this imperative, rather than as a right, it can be seen as an interest. Groll sees rights as a conclusion to the argument about donor anonymity rather than as a premise. He points out that using the language of ‘interests’ allows the exploration of what might be important to people and from that, a discussion can be had of whether those interests are sufficiently weighty to merit seeing them as a right, a right that could impose duties on others. Groll argues that donor conceived people are likely to have a ‘significant interest’ in knowing who the donor is, and that this is an interest that should be furthered on welfare grounds [13].

On the other side of the debate, there are those who argue that there are potential harmful consequences of having identity-release donation programmes. Argumentation used by ethicists who are sceptical about the trend for identity-release gamete donation, such as Guido Pennings and I Glenn Cohen, often draw attention to the practical implications of removing anonymity, such as the reduction in gamete donor numbers that could threaten the viability of reproductive donation programmes, and the potential implications of such a shortage in donors for potential parents. Cohen makes the argument that mandating identity-release, donation would restrict the reproductive autonomy of those who want to reproduce, because it would both determine the type of donor they had to have, identity-release, and reduce their options due to lack of donors, and even the possibility of having treatment with a donor at all [33, 34].

On principled grounds, the argument for anonymity rests on not seeing knowledge of genetic origins as an important human right that must be upheld. For Pennings, knowing who the donor is, is not a fundamental human right, and if it is a right, it is one among many, that has to be balanced against other peoples’ rights, such as the rights of parents and donors [15]. For those who are sceptical about the utility of identify-release donation, such as Pennings and Cohen, information on genetic origins is just not that important. Hence, it can be balanced against other considerations, such as practical matters like reduction in donor numbers, and other parties’ rights, such as the parents’ or donors’ right to privacy.

In conclusion, while there has been significant debate in the literature on the benefits and harms potentially produced by different forms of donation, with appeals to empirical evidence and critiques of such appeals [49, 50], the debate is underpinned by the view taken on the importance given to the knowledge of genetic origins. To my mind this is the nub of the information giving in donor conception debate: what status and importance is given to the claim that donor conceived people have a right and/or interest in knowing their genetic origins. If this is seen it as important, as a human right for example, then identity-release donation is preferred, if it is not, then anonymous donation is perfectly acceptable. Further, if identity-release donation is seen as the ethical way to organise the practice on principled grounds, then the potential practically harmful consequences, such as a fall in donor numbers, will not be seen as decisive arguments. The stance taken on these questions influences the debate over disclosure of donor origins. I will now give an overview of DTCGT and how it is used by those involved by donor conception.

### Direct to consumer genetic testing (DTCGT)

DTCGT use has grown substantial in recent years. The global market was valued at approximately US$1.9–2.0 billion in 2023–2024 and is projected to reach between US$ 8.6 billion by 2030 [51]. In the US alone, the market exceeded US$1.9 billion in 2024 [52]. While North America is the main area of growth, significant expansion has taken place across Europe and Asia–Pacific regions, particularly China and Japan. In the UK, the market is growing rapidly, with projections of 29.3% annual growth through 2030 [51].

The process of DTCGT is reasonably simple, you order a kit online and submit a DNA sample, usually by doing a cheek swab, mail this back to the company and then receive a snapshot of your genetic information via their online portal. The predominant testing methodology employs autosomal DNA analysis, is capable of detecting genetic relationships extending to second cousins and beyond, though accuracy diminishes with more distant connections. DTCGT sites typically position themselves as tools for personal discovery, offering insights into genealogical heritage, family connections, and health predispositions. The information received depends on the DTCGT company, some provide medical information such as propensity to certain diseases and others genealogical data. Leading companies, such as Ancestry.com, integrate genetic analysis with traditional genealogical resources, including historical records and demographic data, to facilitate both contemporary and ancestral family connections.

A key feature across the major platforms is the genetic matching algorithms that connect users with potential relatives within their databases. Using these sites: donor conceived people have found out they are donor conceived; others have searched for the donor and other donor relations, identifying half-siblings; and donors have been contacted by offspring conceived decades earlier [53]. These discoveries can have significant emotional consequences, and challenge established family narratives and personal identities and expose long-held family secrets [23]. The increasing sophistication of DTCGT and the growth in their use have dramatically altered the landscape in which donor conception operates. I will now consider the ethical and regulatory challenges that DTCGT poses for those involved in donor conception.

## The complete removal of gamete donor anonymity

DTCGT has the potential to remove the possibility that donors can be anonymous to their donor offspring at any point after the birth. While identity release programmes allow the donor’s identity to be released at a predetermined time point, such as the age of majority, with DTCGT a donor’s identity could be found at any time. This is a radical shift from previous ways of how information giving occurred in donor conception. It has prompted the UK regulator, the Human Fertilisation and Embryology Authority (HFEA), to propose that, ‘The Act should be amended to enable the removal of donor anonymity from the birth of any child born from donation’ [8, 9]. DTCGT does not so much herald the end of donor anonymity, as many jurisdictions have now mandated identity release programmes, rather it disrupts practice and donor anonymity cannot be guaranteed at any point after the birth of a child.

The claim that DTCGT has precipitated the end of gamete donor anonymity is not new, authors such as Borry and Harper [54, 55] have noted this over 10 years ago. The expanding user base and international reach of these sites can enable connections to be made and this is becoming more likely. Further, it is possible to identify the donor without the donor being registered on a site, as a member of the donor’s family may be registered and the donor can be found through them [56]. Despite this, it is important to bear in mind that the likelihood of finding donors, donor siblings or other donor relations via DTCGT should not be overstated. It is not certain who will be found and some donor conceived people searching for donor relatives via DTCGT are not successful [3]. This in itself can create challenges for those searching, leading to disappointment and lost hope.

However, despite these caveats, DTCGT challenges the whole notion of donor anonymity, which was premised on the promise to donors that they would be able to remain anonymous to any people conceived from their donation in perpetuity. DTCGT, by making donor anonymity impossible to guarantee at any point, has radically changed the context in which donor conception operates. This, arguably, results in anonymous donation programmes becoming unfeasible and this is a strong practical argument for why such programmes should no longer be offered. Pennings [15] disputes this and argues, ‘it is clear that donor anonymity can no longer be guaranteed by the clinics or gamete banks. However, this fact has no implications for the right to know and neither does it lead to the conclusion that anonymity should be abolished.’ (2021, 165–166) He uses the analogy of speed limits, if the speed limit was set at 100 miles per hour at a time when few cars could drive that fast, now that faster cars exist, we would not think of raising the speed limit or claim there is a right to drive faster. This analogy is problematic, as speed limits can still be enforced, the problem is precisely that donor anonymity *cannot* be enforced in the era of DTCGT. While DTCGT in itself does not speak to the acceptability of any way of organizing reproductive donation, it has made donor anonymity practically problematic and, in many cases, impossible. I would argue that this, together with the ethical arguments against donor anonymity, strongly suggests we should no longer have anonymous gamete donation programmes.

## Challenges for regulators—circumventing ‘formal’ systems of information holding and provision

‘The issue of accessing donor information and identifying donors, has become more urgent with the growing popularity of easily accessible, relatively affordable direct-to-consumer DNA testing and matching services which have revolutionised our ability to find our genetic relatives’ [8].

The possible removal of donor anonymity by DTCGT presents challenges to regulators. DTCGT enables people to circumvent regulatory prohibitions on providing information about the donor and donor siblings to the donor conceived person. For countries that mandate anonymous donation, such as Spain, DTCGT has the potential to make such laws redundant. In countries that mandate access to information at an age of majority, such as the UK, DTCGT can be used to circumvent this age restriction. As the UK regulator, the HFEA has noted, ‘The Act’s framework of anonymity as default with managed information release via the HFEA risks being overtaken by technological change. It gives assurances to donors, donor conceived people and their parents that their status in relation to donor conception, their identity and other information will stay confidential until the legal framework permits it to be disclosed, while in reality, the independent exchange of relevant information can freely and legally take place outside of the Act’s control’ [57]. Queensland, Australia, in the report that formed the basis for their law reform, giving donor conceived people the right to identifying information about their donor, mentions the significance of DTCGT and how anonymity can no longer be guaranteed [58].

Due to these concerns, DTCGT has prompted regulators to consider regulatory reform. For example, the HFEA, has proposed that clinics should be required by law to inform donors and recipients about the potential of the donor’s identity being discoverable by DTCGT [8]. While DTCGT is not the only factor prompting law reform, regulators are recognising that they have to respond to the challenges it raises. How regulators might do this is beyond the scope of this paper, see Redhead & Frith [59], but a response is needed or regulation, where it exists, risks becoming sidelined and hence ineffective.

## Challenges for donor conceived people

### Finding out that you are donor conceived via a DTCGT

One potential consequence of DTCGT is that people may find out that they are not genetically related to one of their parents, usually misattributed paternity. Misattributed paternity maybe due to a variety of reasons, extramarital affairs, adoption, etc. But it can also be a result of undisclosed donor conception. Finding out about donor conceived origins via a DTCGT can be emotionally challenging for donor conceived people [3]. Depending on their age, family situation and attitudes of their parents and family, it has the potential to disrupt or trouble existing family relationships. Hence, finding out ‘accidently’ via DTCGT can be problematic for some donor conceived people [23]. In a study that looked at donor conceived people finding out they were donor conceived ‘by accident’ (some had used DTCGT but not all), donor conceived people often felt that it was the keeping of the secret that hurt them the most rather than finding out they were donor conceived [60]. It could also be argued that the donor conceived person, did not consent to receiving this information. They took a DTCGT to find out about their health or distant ancestors and were not expecting to receive information of this type or significance. Further, donor conceived people are left to digest this information, that they did not expect or potentially want, with little support or help.

### New gatekeepers of information

DTCGT can create new ‘gatekeepers’ of knowledge about people’s donor conceived origins and people related them through donor conception. Information about donors and recipients has, up till now, been held by fertility clinics and/or a national register (if one exists in that jurisdiction). Formal systems are operated by trained professionals – fertility clinic staff or those who work for a regulatory authority such as the HFEA in the UK. These professionals follow established legal frameworks and/or relevant professional good practice guidelines, such as those issued by the ASRM [61], and organisational protocols when sharing information about donor conception with the relevant parties. Hence, these systems provide clear guidelines regarding the circumstances, timing, and methods for information giving. In contrast, as an informal system, DTCGT lacks the structured oversight of formal systems, and information becomes known by those who may not appreciate its significance or know how to manage its dissemination appropriately. For example, Person A who is donor conceived may match with a half-sibling, Person B, and realise that Person B is a half-sibling through gamete donation, but Person B is unaware they are donor conceived. Or a donor may match with their donor offspring, and the donor offspring is unaware of their donor conception status. An information asymmetry exists; the donor half-sibling and the donor possess knowledge about an individual’s donor conceived status that the individual does not possess themselves. Hence, information about donor conception origins might become known by various people, the donor, other donor conceived people, or relatives of the donor, who then become gatekeepers of this knowledge. These gatekeepers possess neither professional training nor, in some cases, have direct experience of donor conception, and they may have limited understanding of the complexities and sensitivities involved.

The ConnecteDNA project found that donor conceived participants using DTCGT frequently found themselves in this unexpected role of information gatekeeper. They would recognise the likelihood that the person they had matched with was also donor conceived or potentially a child of their donor, but did not know what knowledge, if any, this person had about their donor conceived status or their parent's donation history [3]. Donors who registered with DTCGT platforms had similar experiences. Some donors had joined DTCGT sites to increase their accessibility to their donor conceived offspring who might want to establish contact. However, many had not anticipated the possibility of matching with people who were unaware that they were donor conceived.

This gatekeeper position presents an ethical challenge for those using DTCGT. The ConnecteDNA study found that participants were unclear what was the right course of action. Did they, the gatekeeper, tell people that they thought they were donor conceived, or did they withhold this information? Participants frequently questioned their choices, particularly when sharing information had caused the person distress, as they felt partially responsible for this. On the other hand, some donor conceived people who had decided not to tell their genetic matches that they were related through donor conception often felt guilty and anxious about their decision to withhold this information. People were put in a difficult position and one they had not anticipated when registering on these sites. DTCGT sites do not adequately signpost possible unexpected consequences, such as misattributed paternity, or becoming gatekeepers of other’s donor conception stories, they are sold as ‘fun’ tools of personal discovery. Hence, people may be unaware of the potential wider consequences of taking a DTCGT. This, arguably, puts some form of obligation on DTCGT companies to support people in these situations, and I will discuss this in the recommendations section.

## Challenges for donors

There are two normative questions that DTCGT poses for gamete donors, should they join a DTCGT site and should they make their genetic data available to the donor conceived person?

### Should donors join a DTCGT site

It might be argued that donors should take steps to make themselves available to be found by their donor offspring and take a DTCGT and, as mentioned above, some donors do join DTCGT sites to do just that [62, 63]. Authors such as Velleman [31] and Groll [13] for example would argue, albeit on different grounds, that knowledge of one’s genetic relations is important, therefore it is advantageous for donor conceived people to be able to find their donor. Given this, it might suggest that gamete donors should seek to help donor conceived people and take proactive steps to make themselves available on DTCGT sites. It could also be argued that donors have some duties or obligations towards their donor conceived offspring, and making themselves available on a DTCGT site could be one way of fulfilling these. However, there are problems with the claim, that donors should do things to benefit their donor-offspring. Even potentially highly valuable goods, such as knowledge of genetic relatives, might not motivate a duty to join a DTCGT, a duty to do something positive for the donor conceived person. A donor might have a moral reason to respond in a kindly fashion if contacted via a DTCGT site, but arguably not a duty or obligation to seek out ways to benefit any donor conceived offspring they may have. What grounds any duties a donor might have to the donor conceived person needs further argument (see Frith: Duties of gamete donors, forthcoming), but while some donors do take this route and made themselves available, it would need further justification to make the case that they were under some obligation to do so.

There are, in addition, some practical arguments against donors making themselves available on DTCGT sites. As has been discussed, there are risks involved in becoming gatekeepers of knowledge of other’s donor conception status and possibly having to tell people that they are donor conceived. There are other routes for donors to make themselves available, without incurring any of the potential problems of using DTCGT. Other options might include, in the UK if you donated anonymously between 1991–2005, you could opt to make your donation non-anonymous by contacting the HFEA. Or joining searching organisations like the Donor Sibling Registry that operate internationally and make links between donor relatives. Options like this ensure that all parties have consented to joining and finding information about their donor relatives, and the unanticipated surprise element of such discovery potentially present with DTCGT is mitigated.

### A donor’s duty to provide genetic data

Genetic data provided by DTCGT companies can be downloaded and shared. For example, on the GEDmatch site you can upload your DNA data from most of the main commercial DTCGT sites. Some DTCGT companies such as My Heritage [64] are now offering whole genome sequencing as part of their testing offer. This data might be useful in the future for the donor conceived person, so that they have additional information about the donor’s medical history and this could be shared by the donor with their donor-offspring or with recipient parents. Shkedi-Rafid et al. [65] have considered the issue of donors sharing their genetic data with recipient parents or the donor offspring in a fertility clinic setting. Shkedi-Rafid et al. conclude that there are ethical difficulties with such sharing: getting meaningful consent from the donor, the donor’s privacy and potential conflicts of interests between the donor and recipients/donor offspring. To ameliorate these, they suggest clinics should act as ‘good-faith brokers’ holding the donor’s data at clinic level and instituting a strict access policy, so this can be controlled and the donor’s data protected. With genetic data that is held on DTCGT sites, there is no-one to act as a broker for this information and the donor has the option to either provide their data if requested or refuse to do this, access cannot be tailored or partial. Again, like the question above, should donors go on DTCGT sites, it is a matter of what, if any, duties or obligations we think donors have towards the donor conceived person. It has been argued that we might not have an obligation to provide our full genome sequence to those children we are bringing up and in a legal parental relationship with [66]. Hence, it seems implausible that the donor should be under an obligation to provide this information to their donor offspring, as this information is highly personal data. However, one might argue that donors have moral reasons to respond to health questions and provide a medical history for their donor offspring if requested, as this seems a reasonable non-onerous request.

Whether donors have any obligations to make themselves available on DTCGT sites and indeed explore other avenues that could enable donor conceived people to find them, or to provide their full genomic data, is contentious. Even if it could be argued that donors have some form of duty or obligation to their donor offspring, it is unlikely that a plausible argument could be made that these include maximal duties—doing everything in one’s power to benefit them [67]. Thus, if donors want to take such steps they can, but they are under no obligation to do so.

## Challenges for parents by donor conception

DTCGT also poses normative questions for parents by donor conception, relating to disclosing donor origins and if and when to test their child.

### DTCGT and parental disclose of donor origins

As noted, DTCGT has created a new landscape in which donor conception operates introducing new risks and benefits that must be weighed in existing discussions. DTCGT potentially affects both the imperative to disclosure and considerations of when to disclose the child’s donor conception origins. As I have discussed in previous papers [45, 46], if donor conceived people have a human right to know their genetic origins (and one that is legally enforced in some jurisdictions), there is a prior right, a right to be told that they are donor conceived, that needs to be fulfilled before donor conceived people can be in a position to access information about their donor. This is generally seen as a moral right, based on basic human rights, that places a duty on the parents.^2^ As Ravitsky notes part of what she calls the right to know (one’s genetic origins), is made up of four rights and one of these ‘is the right to the parental disclosure aspect [that] relates to the right to know the truth about the circumstances of one’s conception as trumping parents’ right to privacy.’ [26], 688).

The importance of disclosure is, as many issues related to information giving in donor conception, based on the significance that is given to the right/interest in knowing one’s genetic origins. Groll [13], as noted earlier, argues that donor conceived people have a significant interest in knowing their genetic origins and this is an interest that parents should reasonably try and facilitate. They should do this by using an open, identity-release, donor, and disclosing to their offspring that they are donor conceived. I will accept this argument for Part I of this section, that this information is important for (some) donor conceived people, and disclosing donor origins is morally desirable. In Part II, I will go on to consider how DTCGT affects the imperative to disclosure even if it is not accepted that disclosing donor conception origins is morally desirable.

#### Part I—Disclosing is morally desirable

I will argue that there is an even greater moral imperative to disclose to the donor conceived person that they are donor conceived, given the existence of DTCGT. One way of approaching an assessment of disclosure is the potential benefits or the harms of disclosing or not disclosing [69]. Circumstances such as DTCGT will affect the benefits or harms produced by disclosure, or forgone and incurred by not disclosing. For example, if there is an identity-release donation programme, by not disclosing to the child they are donor conceived, the child is potentially deprived of exercising their right to access identifying information about their donor when they reach majority. Now, rather than just knowing the identity of the donor, there is the potential of finding and making contact with not only the donor but donor siblings, which some donor conceived people are more interested in than their donor [32], and wider donor relations. With DTCGT by not disclosing the parent is potentially depriving the donor conceived person of the opportunity to exercise their significant interest in finding their donor relatives. Therefore, the benefits of disclosure are now weightier than they were previously. A donor conceived person might have an even greater interest in knowing that they are donor conceived, as this knowledge has the potential to produce more benefit, finding donor relatives, than before such technologies existed.

#### Part II—Disclosing is not morally desirable

The claims that donor conceived people have a significant interest in knowing their genetic origins and hence their donor, are not accepted by all. As discussed, authors such as Pennings and Cohen do not see this information as constituting a significant interest in the way Groll does or that it is a human right. Pennings [50] further argues that there is no empirical evidence that donor conceived people are harmed by not knowing they are donor conceived, or by extension not having access to identifying information about the donor. So, accepting this view for the purposes here, there are still arguments for disclosure that are prompted by DTCGT.

The advent of DTCGT makes it possible that donor conceived people can find out they are donor conceived and this is more likely than ever before, and therefore parental secrecy becomes even more unsustainable. As, discussed earlier, studies on secrets in donor conception [23], demonstrate that accidently finding out about donor origins without being told can be painful. Hence, if the likelihood of finding out about donor origins has increased, and it has been recognized that finding out ‘accidentally’ can be particularly harmful to donor conceived people, then this is another argument for disclosure. It avoids unnecessary harm for the donor conceived person. It might be objected that this is only a practical argument for disclosure and not a normative one, in that it points only to the possible bad consequences of not disclosing. However, many of the arguments against identity-release donation, that it will reduce the supply of donors, and restrict recipients’ choices and limit reproductive autonomy, are practical ones, and such practical concerns have normative consequences.

In sum, the circumstances in which donor conception operates dictate how important disclosure is. It could be argued that the greater ability to find the donor, donor siblings and wider donor relatives afforded by DTCGT mean that there are arguably greater benefits for donor conceived people in knowing that they are donor conceived and greater disbenefits in being denied this knowledge. This adds weight to the claim that parents should disclose donor-conception origins to their donor conceived offspring. In the practice literature about disclosing donor conception origins, there is a consensus that telling children as early as possible, as soon as they are likely to understand, is optimal [30], and some evidence that late disclosure or accidental finding out is sub-optimal [49, 70].

### What age should recipient parents test their donor offspring?

Most jurisdictions that mandate identity-release donation stipulate that the donor conceived person must reach the age of majority before they can access identifying information about their donor. With DTCGT these age restrictions can be circumvented. While most platforms specify minimum ages of 13–16 years for general use and 18 years for purchasing test kits [71], the practical reality is very different. On DTCGT sites parents can set up profiles for their children below the age of majority and, if they wish, test them at a very young age.

DTCGT raises the following questions for parents of donor conceived offspring. Should parents test their child at a very young age, arguably before the child can provide meaningful consent, so that the child might ‘grow up with’ donor-siblings, or have knowledge about the donor and donor relatives? Alternatively, should parents adopt a more cautious approach and support their child’s use of DTCGT only if and when the child independently expresses interest in doing so and can consent to this? If pursuing this latter approach, what level of understanding should the child demonstrate, regarding, for example, the potential challenges involved in connecting with donor relatives or the broader implications of online data sharing, before parents support this choice? Or should parents attempt to enforce any official age restrictions that operate in the jurisdiction in which they live? The ConnecteDNA study explored recipient parent’s experiences of using DTCGT and found different views on what was an acceptable age to test their child. Parents felt they were faced with a moral dilemma, there were equally compelling reasons for not testing or testing, and there was little consensus among the participants on which was the right option [63].

The resolution of this dilemma is unclear; there are persuasive arguments for both early and later testing. Early testing enables the child to potentially know and grow up with their donor siblings and form bonds from early childhood. However, getting to know and having contact with the donor might be more fraught and difficult when donor conceived people are young children. Later testing could be supported on grounds that it is best to wait until the child is sufficiently mature to make that decision themselves, and they can give informed consent to the test. There is nothing in principle wrong with parents choosing for their child, parents regularly make medical and life-changing decisions for their children (which school, where to live etc.). Not testing could represent forgone opportunities for the child to interact with their donor relatives, particularly donor-siblings when the child is young. However, any option, testing and not testing, closes off options and open others that cannot be undone. In making this difficult decision it is important that parents are well-informed about the potential long-term consequences of their decision. These might be the implications of data sharing and how they might feel about interacting with donor relatives when their child is young, both in terms of the child’s needs and their own.

A further consideration is that donor conceived children, under the age of majority, could potentially, access these sites without parental knowledge. This concern was discussed by parents in the ConnecteDNA study, who knew that donor conceived teenagers might be able to access DTCGT services independently, potentially without their knowledge. The enforcement of the age restrictions for the use of DTCGT can be ineffective in practice and DTCGT companies should consider if they need to take measures to address this.

## Recommendations

I conclude the paper by making some recommendations for how the ethical challenges posed by DTCGT might be, if not overcome, at least ameliorated. First, DTCGT companies need to provide more information and better signposting about the possible wider implications of using their services. For example, the possibility of finding unexpected genetic relations and not being genetically related to immediate family, particularly misattributed paternity, needs to be clearly mentioned. Additionally, DTCGT sites need to provide better information about how these tests can affect donor-conceived individuals, adoptees, and other groups who might find they are not genetically related to those bringing them up [72]. Second, fertility clinics need to consider how they can inform and support recipients and donors. Those undergoing fertility treatment and prospective donors should be informed about DTCGT at the time of treatment (see [73]), with the full implications of this technology clearly explained. Donors should be made aware that their anonymity cannot be guaranteed and understand the longer-term implications of donating. For example, a donor may be contacted by their donor offspring many years after donating and therefore these implications are not temporally limited. Recipient parents need to be aware of the implications of DTCGT for how they approach disclosure of their child’s donor conceived status and how to prepare for making a decision over whether they will test their child when they are young. Generally, greater information provision and awareness of the potential issues will help people involved in donor conception be cognisant of DTCGT and, if they use these services directly, have adequate knowledge of all the potential ramifications. However, simply providing information does not ‘solve’ the ethical challenges created by DTCGT and it might be argued that stricter regulation of these sites is needed. Given the multinational operation of DTCGT companies, this would be hard, although not impossible, to achieve and what good regulation of this area should look like needs further exploration. Third, greater support is needed for the donor conception community. Many of the people most affected by using DTCGT will not be in touch with fertility clinics or any other healthcare professionals and hence, there is a need for other kinds of support for those affected. What support is appropriate and feasible will depend on the particular setting and will vary between jurisdictions and healthcare systems [74]. Work needs to be done in different settings to explore how support can be provided and made accessible to those who need it.

Finally, regulators of fertility treatment need to consider how best to respond to this new landscape in which donor conception operates. This paper has not considered regulation in any detail (see [59]). The point here is to emphasize that regulators and those that produce guidelines need to be aware of how DTCGT affects donor conception and to take this into account when formulating regulations, policies and good practice recommendations.

## Conclusion

The central purpose of this paper has been to draw attention to the ethical challenges DTCGT poses for the donor conception community. This paper considered the challenges raised for: regulators, donor conceived people, donors and parents by donor conception. Perhaps the most significant implication of DTCGT is making anonymity (almost) impossible to guarantee at any point for the donor. This has radically changed the context in which donor conception operates and anonymous donation programmes have become practically unfeasible. This practical reality, coupled with the ethical arguments against anonymous donation, provide compelling grounds for discontinuing such programmes. The paper concluded by considering how the challenges posed by DTCGT might be ameliorated, focussing on the importance of information provision for all those involved. While this will not solve the ethical challenges it will at least ensure that those who use DTCGT do so equipped with adequate knowledge to navigate this complex environment. DTCGT companies need to take responsibility for some of the unintended consequences of using their services and both signpost information and provide support for those who may be adversely affected. Fertility regulators must adapt to this new landscape or risk obsolescence, with their regulations rendered ineffective and their authority undermined.

## Data Availability

Data available on UK Data Service ReShare (https://urldefense.com/v3/__https:/reshare.ukdataservice.ac.uk/858176/__;!!PDiH4ENfjr2_Jw!BoxJZh7wDWqb1HnbMcxru7RARmiIGfp1kpcGkvlcIpnbWhSUy4NXwVlFVp7y3HhPoWNUl0JsZxFmf2n7R5TzG_v0k2A7PROkmso).

## Footnotes

1 Due to the increasing use of DTCGT and other technologies that make it easier to identify donors, the ASRM in 2022 recommended that the terms known donor and anonymous donor be replaced by directed donor and non-identified donor.

2 Most legislatures have been reluctant to turn this moral right into a legally enforceable right to be told of your genetic origins. However, in 2019 Sweden passed a regulation that obligated parents to inform their children that they are donor-conceived [68].
